# Too young for Cannabis? Choice of minimum legal age for legalized non-medical Cannabis in Canada

**DOI:** 10.1186/s12889-020-08639-z

**Published:** 2020-05-14

**Authors:** Hai V. Nguyen, Stephen Bornstein, John-Michael Gamble, Maria Mathews, Lisa Bishop, Shweta Mital

**Affiliations:** 1 School of Pharmacy, Memorial University of Newfoundland, 300 Prince Philip Drive, St. John’s, NL A1B 3V6 Canada; 2 Faculty of Medicine, Memorial University of Newfoundland, St. John’s, Canada; 3 Director, Newfoundland and Labrador Centre for Applied Health Research, St. John’s, Canada; 4 School of Pharmacy, Faculty of Science, University of Waterloo, Waterloo, Canada; 5 Department of Family Medicine, Schulich School of Medicine & Dentistry, Western University, London, Canada

**Keywords:** Minimum legal age, Cannabis legalization, Canada

## Abstract

**Background:**

Choice of minimum legal age (MLA) for cannabis use is a critical and contentious issue in legalization of non-medical cannabis. In Canada where non-medical cannabis was recently legalized in October 2018, the federal government recommended age 18, the medical community argued for 21 or even 25, while public consultations led most Canadian provinces to adopt age 19. However, no research has compared later life outcomes of first using cannabis at these different ages to assess their merits as MLAs.

**Methods:**

We used doubly robust regression techniques and data from nationally representative Canadian surveys to compare educational attainment, cigarette smoking, self-reported general and mental health associated with different ages of first cannabis use.

**Results:**

We found different MLAs for different outcomes: 21 for educational attainment, 19 for cigarette smoking and mental health and 18 for general health. Assuming equal weight for these individual outcomes, the ‘overall’ MLA for cannabis use was estimated to be 19 years. Our results were robust to various robustness checks.

**Conclusion:**

Our study indicated that there is merit in setting 19 years as MLA for non-medical cannabis.

## Background

Choice of minimum legal age (MLA) for cannabis use is a critical and contentious issue in non-medical cannabis legalization. In Canada (which legalized non-medical cannabis in October 2018), the federal government task force on cannabis legalization recommended an MLA of 18 years [1], stating it chose a low MLA to discourage the persistence of the underground market [2]. Following public consultations and for harmonization with the existing MLAs for alcohol and tobacco, Canadian provinces – which were allowed to choose a higher MLA than the federal recommendation -- set 19 as the MLA for non-medical cannabis use; the two exceptions were Quebec and Alberta which opted for 18 as MLA [3].

Meanwhile, critics had argued for a higher MLA of 21 or even 25 years citing evidence that brain development does not reach completion until early adulthood and that before then, the brain is particularly sensitive to damage from drug exposure [4, 5]. In addition, longer education, delayed marriage and parenthood among present-day youth imply delay in achievement of adulthood [6]. For comparison, all US states that have legalized non-medical cannabis (i.e. Alaska, California, Colorado, Illinois, Maine, Massachusetts, Michigan, Nevada, Oregon, Vermont and Washington, and the District of Columbia) opted for an MLA of 21 [7].

Even though MLAs have been adopted by the provinces, the ongoing debate suggests that the situation around the policy on minimum legal age for cannabis use in Canada is still dynamic and evolving. A national poll indicated that nearly half of all Canadians prefer the MLA to be above 20 [8]. There have also been calls to raise MLA to 21 in Ontario [9]. In particular, Quebec recently revised its MLA to 21 in October 2019 [10], which will likely prompt further debates and re-evaluation of existing MLAs in other provinces.

Notably, evidence to shed light on the merits of these different specific ages as MLAs is still limited. Neuroscientific evidence suggests that the pre-frontal cortex continues to develop into early adulthood and exposure to cannabis in adolescence results in executive dysfunction, attention deficits, reduced overall intelligence, cognitive inhibition and abstract reasoning, and abnormal brain activation [11]. Further, earlier age of cannabis use onset is associated with higher frequency and amounts used; the latter are crucial in determining the degree of cannabis related impairment [12]. While this body of neuroscience research on impacts of cannabis use is important, evidence on the severity and persistence of cannabis -related cognitive impairment is inconclusive [13]. Further, such evidence may not be sufficient to inform policymakers who often work with socio-economic outcomes. Meanwhile, although several studies have looked at the relationship between age of first cannabis use and educational outcomes [14–17] and smoking behaviors [18, 19], none of these studies explicitly assessed these outcomes at different specific ages of first cannabis use to inform merits of different MLAs. The objective of this study is to fill this evidence gap to inform the continued debate over the optimal MLA for cannabis in Canada and to guide policymaking in jurisdictions planning to legalize non-medical cannabis in the future (including Mexico, New Zealand, Russia, Luxembourg and several US states [20]). Specifically, we estimate and compare educational attainment, cigarette smoking, self-reported general and mental health outcomes associated with different ages of first cannabis use that are being discussed as potential MLAs, namely, 18, 19, 21 and 25 years. We do not consider potential MLAs lower than 18 or higher than 25 years of age because these ages are neither being considered in policy discussions nor practical (an MLA under 18 would be too low while an MLA above 25 would encourage a large underground market).

## Methods

### Study design and data source

This population-based cross-sectional study used data from multiple waves of the nationally representative Canadian Tobacco Use Monitoring Surveys (CTUMS) conducted between 2004 and 2012 and its subsequent version, the biennial Canadian Tobacco, Alcohol and Drugs Survey (CTADS) conducted in 2013 and 2015. The CTUMS employs a cross-sectional stratified survey design and annually interviews a nationally representative sample of nearly 20,000 individuals aged 15 years and older. The survey’s response rate has been high, about 83% [21]. Survey weights are estimated and placed on each record to represent the number of sampled persons that the record represents. The CTADS biennially interviewed a nationally representative sample of nearly 15,000 Canadians aged 15 years and older on their use of tobacco, alcohol and illicit drugs. Both the CTUMS and the CTADS oversampled adolescents and youth, facilitating the focus of our analysis on these age groups.

### Variables

Our choice of outcome measures was guided by the potential adverse impacts of cannabis on socio-economic, behavioral and health outcomes documented in the literature. Specifically, there exists evidence that early cannabis initiation is associated with higher school dropout rates, greater substance use and poor health outcomes later in life [18, 22–24]. Accordingly, we studied educational attainment, current cigarette use and self-reported general and mental health. While some studies have assessed the impact of cannabis initiation on employment outcomes in early adulthood [25], there is limited evidence on the impact on employment outcomes in later life, which is the focus of this study. Hence, we did not assess employment as an outcome.

Data on educational attainment were available only in the CTUMS while data on current cigarette use were obtained from both the CTUMS and the CTADS. Data for self-reported general and mental health were available only in CTADS. Education attainment was derived based on responses to the question: “*Highest level of education: no schooling, completed elementary, some secondary, completed secondary, some community college, completed community college, some university, completed university or other education and training*?” Specifically, we constructed an ordinal six-level variable to represent educational attainment: 1. Less than secondary (no schooling, completed elementary, some secondary); 2. Completed secondary; 3. Some community college; 4. Completed community college; 5 Some university and 6. Completed university. A respondent was classified as a current cigarette smoker if s/he reported having smoked cigarettes either every day or occasionally at the time of the survey. Self-reported general health was based on the survey question: “*In general, would you say your health is...?”*: *(Excellent, Very Good, Good, Fair, Poor)* while self-reported mental health was based on responses to the question: “*In general, would you say your mental health is...?”*: *(Excellent, Very Good, Good, Fair, Poor)*. As the number of respondents who reported ‘poor’ or ‘fair’ general or mental health was small, we constructed a four-level ordinal variable to represent these outcomes with higher values indicating better outcomes: 1. Poor or fair; 2. Good; 3. Very Good; 4. Excellent.

The independent variable of interest was self-reported age of first cannabis use. As we focused on the four specific ages being discussed as potential MLA (18, 19, 21 and 25), we divided age of first cannabis use into four groups for comparison: < 18, 18, 19–20 and 21–24 years.

### Study sample

Our study sample included respondents aged between 21 and 65 years. At a younger age than 21, most people may not yet attain the highest level of education. Beyond 65, the effect of age of first cannabis use may wane off and other factors may play a greater role in explaining behavioral and health outcomes.

We excluded respondents who initiated cannabis at or after age 25 because, as noted earlier, an MLA higher than 25 is not practical. We also excluded respondents who used cannabis only once in their lifetime (a proxy for experimental use) as such use is unlikely to have a meaningful impact on later life outcomes. Number of respondents excluded based on each criteria are shown in Fig. A1 in the Online Supplementary Materials.

### Criteria for determining MLA

We note that the MLA for different outcomes could be different. An age is chosen as the MLA for an outcome if it satisfies three criteria. First, the outcome for first cannabis use at that age or later is significantly better than the outcome for first use at earlier ages (if not, a lower age could be the MLA). Second, the outcome for first use of cannabis at that age should not be significantly worse than the outcome for first use at later ages (if not, a higher age can be the MLA). Finally, the chosen age should be the lowest among all potential MLAs. For instance, if outcomes of first cannabis use at age 19 and age 21 are significantly better than before age 18 but are not significantly different from each other, then age 19 would be chosen as MLA. (As we cap the maximum MLA at 25, we will only choose age 25 as MLA if all the three criteria above are not satisfied. This can happen, for example, when outcomes of first cannabis use at any later age (between age 18 and age 24) are not significantly better than at any younger age). Assuming each outcome carries an equal weight, an ‘overall’ MLA is calculated as the average of the MLAs for the individual outcomes.

### Statistical analysis

Descriptive analysis was conducted to examine demographic characteristics of the full study sample and of each of the four age groups of first cannabis use. F-tests were conducted to compare these characteristics across the four age groups of first cannabis use.

We use regression analyses to estimate the association of various ages of first cannabis use with the outcomes of interest. To account for potential confounding factors that influence the age of first cannabis use (such as emotional distress, risk taking attitude, or peer influence), we employed two robust estimation techniques, namely, the augmented inverse propensity weighted (AIPW) estimator [26–28] and Marginal Mean Weighting through Stratification (MMWS) [29].

The AIPW estimator combines a propensity score model for the treatment (i.e., age of first cannabis use) using an inverse probability-weighted method and a model for the outcome using a regression adjustment method. The AIPW estimator is doubly robust as it can generate consistent treatment effects whenever either the propensity score model or the outcome model is correctly specified [28]. It has been shown to outperform three popular estimators: a regression estimator, an inverse propensity weighted (IPW) estimator, and a propensity score matching estimator [30]. In this study, the propensity score model was estimated using a multinomial logistic regression. We regressed the categorical variable for age group of first cannabis use (i.e., < 18, 18, 19–20 and 21–24 years) on demographic characteristics (age, sex, marital status, household size, rural/urban status, and language spoken at home), cigarette smoking status, use of other tobacco products and province and year indicators. Province indicators were included to capture any province-specific factors that affect the age of first cannabis use. Year indicators controlled for any nation-wide changes over time as well as any temporal changes across surveys. The outcome model was estimated using a linear regression that included indicators for age groups of first cannabis use and the same set of controls as in the propensity score model. All estimates in the descriptive analyses were weighted using survey sampling weights and robust standard errors were clustered at the provincial level.

MMWS is a recently developed, generalized form of propensity score based estimation technique that can yield unbiased estimates in observational studies [29]. It has been found to be more accurate than other propensity score approaches such as inverse probability weighting [29]. It combines both stratification and weighting based on propensity scores while allowing for multi-valued treatment variables. This method first calculates propensity scores for each observation based on the respondents’ characteristics and assigns a weight to each observation so that the resulting age groups of first cannabis use are balanced on observed characteristics of respondents [29]. Age of first cannabis use < 18 years is the reference category. Coefficient estimates obtained from both AIPW and MMWS estimates reflect the difference in outcome between the respective age group of first cannabis use and this reference category.

We also conducted some robustness checks. First, we utilized the recent novel ‘coefficient stability approach’ developed by Oster (2017) [31] to assess the potential bias of our estimates. This technique uses information on the correlation between age of first cannabis use and observed characteristics of the respondent to predict the relationship between age of first cannabis use and the unobserved characteristics that influence the outcome. In doing so, it provides a range for the estimate for the association between age of first cannabis use and our outcomes of interest. An estimate is considered as robust if its range generated by the Oster’s method does not cross (i.e., does not contain) zero value. Second, we conducted balance tests [32, 33] to check if the observed characteristics of respondents were similar across the four age groups of first cannabis use, after inverse probability weights are applied. These tests involved estimating ‘standardized differences’ as the differences in covariate means (proportions) between respondents in different age groups of first cannabis use, standardized using sample variance (prevalence) of the covariate in each age group [27]. Covariates across different age groups of first cannabis use were considered to be balanced if standardized differences in the weighted sample did not exceed 10% [32]. Achieving balance on covariates across age groups of first cannabis use would ensure that age of first cannabis use was ‘as good as random’ after conditioning on these observed characteristics [33]. Then, any differences in outcomes across different ages of first cannabis use can be linked with such use. Finally, we assessed the robustness of our results to our choice of study sample. First, we expanded the study sample to include one-time cannabis users. That is, we re-estimated the MLA for the study sample that included both who used cannabis once and those who used it more than once in their life. Second, we narrowed the sample to focus only on those who reported using cannabis both more than once in their life and within 12 months preceding the survey.

## Results

### Descriptive statistics

Table 1 presents the demographic, socio-economic and substance use patterns of respondents. Panel A includes respondents from the eight CTUMS cycles and Panel B from the two CTADS cycles. Respondents across the four age groups of first cannabis use were similar both in the CTUMS and CTADS, except that average age of respondents was higher in higher age groups of first cannabis use among CTUMS respondents. Further, respondents in higher age groups were more likely to have completed university and were less likely to be current smokers.

**Table 1:** Summary Statistics

	(1)	(2)	(3)	(4)	(5)	(6)
	Age (21–65 years)	Age of first cannabis use < 18 years	Age of first cannabis use 18 years	Age of first cannabis use 19–20 years	Age of first cannabis use 21–24 years	*p*-value
**Panel A: CTUMS (2004–2012)**						
Education:						
Less than secondary	0.08	0.10	0.06	0.06	0.07	< 0.001
	(0.28)	(0.29)	(0.24)	(0.23)	(0.25)	
Completed secondary	0.37	0.40	0.35	0.32	0.31	< 0.001
	(0.48)	(0.49)	(0.48)	(0.47)	(0.46)	
Completed college	0.25	0.26	0.25	0.23	0.21	0.006
	(0.43)	(0.44)	(0.43)	(0.42)	(0.40)	
Completed university	0.30	0.25	0.33	0.40	0.42	< 0.001
	(0.46)	(0.43)	(0.47)	(0.49)	(0.49)	
Cigarette smoker	0.33	0.37	0.29	0.24	0.24	< 0.001
	(0.47)	(0.48)	(0.45)	(0.43)	(0.43)	
Age	38.93	37.18	40.79	42.03	43.59	< 0.001
	(11.52)	(10.73)	(11.71)	(12.45)	(12.38)	
Male	0.58	0.57	0.61	0.61	0.57	0.018
	(0.49)	(0.49)	(0.49)	(0.49)	(0.50)	
Urban	0.82	0.82	0.83	0.82	0.82	0.724
	(0.39)	(0.39)	(0.38)	(0.39)	(0.38)	
English	0.75	0.75	0.75	0.76	0.80	0.024
	(0.43)	(0.43)	(0.43)	(0.43)	(0.40)	
N	37,244	23,954	5412	5416	2462	
**Panel B: CTADS (2013 and 2015)**						
Current cigarette smoker	0.24	0.28	0.23	0.18	0.12	< 0.001
	(0.43)	(0.45)	(0.42)	(0.39)	(0.33)	
General health	2.84	2.82	2.83	2.80	3.00	0.144
	(0.89)	(0.87)	(0.90)	(0.95)	(0.86)	
Mental health	3.09	3.09	3.00	3.21	3.07	0.072
	(0.88)	(0.86)	(0.94)	(0.84)	(0.94)	
Age	40.83	39.69	43.71	42.16	41.07	< 0.001
	(12.49)	(11.79)	(13.55)	(13.29)	(12.56)	
Male	0.59	0.58	0.62	0.62	0.54	0.306
	(0.49)	(0.49)	(0.49)	(0.49)	(0.50)	
Urban	0.80	0.79	0.81	0.81	0.78	0.708
	(0.40)	(0.41)	(0.39)	(0.39)	(0.42)	
N	6783	3847	1057	1245	634	

Note: Data in Panel A are from CTUMS (2004–2012) and in Panel B from CTADS (2013 and 2015). Values are mean (SD). Sample includes respondents aged 21–65 years who initiated cannabis before age 25. Estimates are adjusted for sampling weights. The variable ‘English’ captures the proportion of respondents who reported speaking English language most often at home

### Regression results

Regression estimates are presented in Table 2. Panel A presents the AIPW estimates and Panel B shows MMWS estimates. Column 1 shows that education level was positively correlated with age of first cannabis use. In particular, education level among those who started cannabis at age 21–24 was significant higher relative to those who started before age 21 (*p* < 0.01), suggesting that the MLA based on educational outcome should be 21.

**Table 2:** Association between age of first cannabis use and later life outcomes

	(1)	(2)	(3)	(4)
Outcome:	Education (CTUMS)	Current cigarette smoking (CTUMS+CTADS)	Self-reported general health (CTADS)	Self-reported mental health (CTADS)
***Panel A: AIPW estimates***				
Age of first use 18 years	0.275***	−0.094***	0.077**	0.044
	(0.026)	(0.009)	(0.037)	(0.034)
	z = 10.5, *p* = 0.00	z = −10.3, *p* = 0.00	z = 2.1, *p* = 0.04	z = 1.3, *p* = 0.20
Age of first use 19–20 years	0.394***	−0.121***	0.057**	0.086***
	(0.020)	(0.010)	(0.025)	(0.023)
	z = 19.9, *p* = 0.00	z = − 11.8, *p* = 0.00	z = 2.3, *p* = 0.02	z = 3.7, *p* = 0.00
Age of first use 21–24 years	0.590***	−0.126***	0.110***	0.068**
	(0.040)	(0.011)	(0.030)	(0.030)
	z = 14.8, *p* = 0.00	z = −12.0, *p* = 0.00	z = 3.6, *p* = 0.00	z = 2.2, *p* = 0.03
***Panel B: MMWS estimates***				
Age of first use 18 years	0.283***	−0.094***	0.070	0.034
	(0.030)	(0.008)	(0.041)	(0.037)
	*t* = 9.36, *p* = 0.00	*t* = 11.47, *p* = 0.00	*t* = 1.72, *p* = 0.12	*t* = 0.92, *p* = 0.38
Age of first use 19–20 years	0.414***	−0.123***	0.067**	0.089**
	(0.023)	(0.010)	(0.023)	(0.029)
	*t* = 17.92, *p* = 0.00	*t* = 12.46, *p* = 0.00	*t* = 2.90, *p* = 0.02	*t* = 3.11, *p* = 0.01
Age of first use 21–24 years	0.630***	−0.141***	0.093**	0.029
	(0.043)	(0.009)	(0.037)	(0.038)
	*t* = 14.55, *p* = 0.00	*t* = 15.33, *p* = 0.00	*t* = 2.53, *p* = 0.03	*t* = 0.77, *p* = 0.46
R squared	0.014	0.013	0.004	0.002
N	35,904	42,610	6598	6593

Note: Data are from CTUMS 2004–2012 and CTADS 2013 and 2015 as indicated in table. Panel A shows the AIPW estimates and Panel B shows the MMWS estimates. Estimates are the difference in outcome for the specific age group of first cannabis use relative to the reference category of age of first use < 18 years. Linear regression models were estimated for all outcomes (including ordered outcomes i.e., education, general health and mental health as the Stata command ‘teffects aipw’ for AIPW does not allow use of ordered logit or ordered probit outcome models.) All models include province and year fixed effects, as well as controls for: respondent’s age, household size, sex, place of residence (urban/rural), marital status and use of tobacco products other than cigarettes). Models in column 1 also control for language spoken at home. All models except column 2 control for respondent’s smoking status. Sample includes respondents with current age 21–65 years who initiated cannabis before age 25. Only respondents who used cannabis more than once in lifetime are included. Standard errors (in parentheses) are clustered at province level. Significance levels are: *** *p* < 0.01, ** *p* < 0.05, * *p* < 0.1

Respondents who first used cannabis at ages 19–20 were 3 and 12 percentage points less likely to smoke cigarettes than those first using at age 18 and before age 18 (*p* < 0.01), respectively. Meanwhile, the likelihood of cigarette smoking among those initiating cannabis during age 21–24 was not significantly different than those who first used cannabis at age 19–20. Hence, for the cigarette use outcome, the MLA would be 19.

Self-reported general health was significantly higher among those who started cannabis after age 18 relative to those who started before age 18 (*p* < 0.05). Further, no significant difference existed among age groups older than 18 (*p >* 0.1). Consequently, the MLA would be the lowest of all potential MLAs, i.e. age 18. Self-reported mental health was higher among those first using cannabis at age 19–20 than before age 18 (*p* < 0.01). This was also not significantly different from the outcome when first using cannabis at age 21–24. Hence, the MLA for this outcome would be 19.

Given our findings of an MLA of 21 for education, 18 for general health and 19 for current smoking and self-reported mental health, the overall MLA for non-medical cannabis use is estimated to be 19, i.e., the average of the four MLAs with equal weights.

Coefficients for control variables in auxiliary equations are reported in Table A1 for AIPW models and Table A2 for MMWS models, and the full set of the contrasts among different age groups of first cannabis use are reported in Tables A3 and A4, respectively (see Section S.2 in Online Supplementary Materials). As shown in the tables, older age and urban status were associated with higher age of first use of cannabis. Coefficients on current cigarette smoking and other tobacco product use were negative and statistically significant for all regressions, indicating that current cigarette smoking and tobacco use are correlated with a lower age of first cannabis use.

### Robustness checks

Table 3 presents the Oster’s bounds for the MMWS estimates. These bounds suggest that our estimates are robust as they exclude zero for all estimates that were found to be statistically significant. Balance tests indicated that none of the standardized differences in the weighted sample exceeded 10% (see Section S.3 in Online Supplementary Materials), indicating that weighting using inverse probability of treatment resulted in balance in covariates across different age groups of first cannabis use [32]. Even when we (i) included one-time cannabis users in our study sample; and (ii) narrowed our sample to include only those who used cannabis more than once and in the past 12 months, conclusions remain unchanged from the base case (see section S.4 in Online Supplementary Materials).

**Table 3:** Robustness checks: Oster bounds

	(1)	(2)	(3)	(4)
Outcome:	Education (CTUMS)	Current cigarette smoking (CTUMS+CTADS)	Self-reported general health (CTADS)	Self-reported mental health (CTADS)
Age of first use 18 years	(0.283, 0.440)	(−0.130, −0.094)	(0.094, 0.132)	(0.049, 0.082)
Age of first use 19–20 years	(0.414, 0.483)	(−0.138, − 0.123)	(0.125, 0.140)	(0.114, 0.129)
Age of first use 21–24 years	(0.630, 0.698)	(−0.156, − 0.141)	(0.164, 0.180)	(0.084, 0.099)
RMax (1.3*R squared)	0.018	0.018	0.005	0.003
Delta	1	1	1	1
N	35,904	42,610	6598	6593

Note: Oster bounds are estimated using the Stata command ‘psacalc’ and are based on Oster (2019). This command can only be performed after linear regressions (commands ‘regress’, ‘aregress’ or ‘xtregress’ in Stata) and thus can only be used for MMWS estimation (which uses ‘regress’ to estimate the outcome model), but not for the AIPW estimation

## Discussion

This study compared educational attainment, cigarette smoking, self-reported general and mental health outcomes across four different age groups of first cannabis use, namely, < 18, 18, 19–20 and 21–24 to assess the merits of the age thresholds of 18, 19, 21 and 25 years being debated in policy discussions as potential MLAs. Our results indicated that, contrary to the Canadian federal government’s recommendation of 18 and medical community’s support for 21 or 25 [34], 19 is the optimal MLA for non-medical cannabis use. This finding is in line with the choice of MLA in most provinces.

Our findings of different MLA for different outcomes warrant discussion. The lower education attainment associated with initiating cannabis before 21 may be a result of poor neurological and cognitive development due to early cannabis use [5]. It is also possible that as the majority of the students complete university around age 21, using cannabis before that age might lead to higher dropout rate [14, 15, 35, 36]. Meanwhile, higher likelihood of cigarette smoking associated with using cannabis before age 19 possibly reflects the greater influence of individual characteristics during adolescence (such as risk taking, sensation seeking and peer influence) [37, 38]. Finally, the MLA of 18 for general health and 19 for mental health may be driven by the possibility that those who initiate cannabis early may be using it as a ‘gateway’ for other illicit drugs [39] which would result in poor health outcomes in later life [40].

Our choice of MLA is supported not only by the statistical significance of the differences between the estimates but also by the magnitude of these differences. Where differences in coefficients between two age groups are statistically significant, these differences are also large in effect size. Conversely, where these differences are not statistically significant, differences in effect sizes are also not economically meaningful. Specifically, for education outcome for which we chose age 21 as MLA, educational outcomes were 16% higher among those who first used cannabis at age 21–24 relative to those who first used it before age 18. Similarly, prevalence of cigarette smoking among those who first used cannabis at age 19–20 was 34% lower compared with the 28% prevalence of cigarette smoking among those who first used cannabis before 18. Meanwhile, general health was 3, 2 and 4% higher among those first used cannabis at age 18, 19–20 and 21–24, respectively, relative to those who first used it before age 18. This justifies the choice of 18 as the MLA for general health. Similarly, for mental health, effect size of first using cannabis at age 19–20 (2.8%) was twice as large as that for first using it at age 18 but was not much different from first using it at age 21–24. This lends support to our choice of 19 as MLA for mental health.

In this study, we assumed that setting an MLA for cannabis use is necessary (which appears a reasonable assumption as most governments set MLAs for substance use) and sought to determine an ‘optimal’ MLA. The choice of an MLA represents a trade-off that policymakers face between curtailing illegal economic activity versus safeguarding adolescents’ well-being. While the medical community recommended an MLA of 21 or 25 based on neuroscientific evidence about adverse impacts of cannabis on cognitive development, this would lead to a large underground market for cannabis. On the contrary, policymakers have decided on a lower MLA such as 18 or 19 to curb the size of underground market, but this raises concerns about adverse outcomes for adolescents. This study, however, found that later life outcomes associated with first using cannabis at age 19 are better than those associated with first using it at age 18 but not significantly different from those first using between 21 and 25. This finding helps to address concerns over potential adverse outcomes associated with setting a low age as MLA. At the same time, our findings can also be used to highlight to youth and their caregivers, the long-term adverse impacts of starting cannabis before age 19.

Our study has some limitations. First, we could not establish the causal effect of age of first use of cannabis on our outcomes of interest. In particular, due to data limitations, we were unable to account for unobserved determinants of current outcomes that may be correlated with age of first cannabis use. However, we employed two robust estimation techniques to account for observed confounding factors and our results were robust to several robustness checks. Further, we studied the relationship between age of first use (which occurred in the past) and current outcomes, which helped rule out reverse causality. We also chose to study the outcomes where there has been moderate to strong evidence that cannabis use does affect these outcomes. Second, as our analysis used pre-legalization data, we were unable to predict the impact of cannabis use post-legalization. Nevertheless, our estimates provide a useful indication for potential impacts of the cannabis legalization. Third, given considerable time lag between survey response and first cannabis use, respondents (particularly, older adults) may not accurately recall their age of first cannabis use. However, previous studies have found that survey responses on age of first cannabis use are considerably reliable [41]. Fourth, we excluded those who used cannabis only once in their life (a proxy for experimental use) in our base case analysis. While it would be more accurate to define experimental use as use of cannabis a few times (instead of once), data limitations precluded such definition. Nevertheless, our sensitivity analyses indicate that our conclusions are robust to alternative definitions of cannabis use. Finally, our study looked at only four outcomes and assumed they carried the same weight in determining an overall MLA. Policymakers might have other important outcomes (such as driving behaviors and street drug use) and/or different weighting scheme when deciding on an MLA. However, the framework we developed in this study can be easily used to accommodate such analyses.

## Conclusions

Our results suggest that there is merit in setting 19 as the MLA for non-medical cannabis use. This finding is consistent with the MLA of 19 currently adopted in most Canadian provinces, but not with the recent MLA increase to 21 in Quebec. Further evidence on health effects of cannabis use and on changes in cannabis use after the cannabis legalization is needed to further inform the policy debate on MLA. In the meantime, it is important to watch trends in youth’s and young adults’ use and monitor harms over time while ensuring that the legal market is tightly regulated and that cannabis companies do not stray from restrictions on marketing, sales, and packaging that might make products more appealing and accessible to children.

## Supplementary information

**Additional file 1.** Supplementary materials.

## Data Availability

Data used in this study are from publicly available versions of Canadian Tobacco Use Monitoring Surveys (2004–2012) and Canadian Tobacco, Alcohol and Drugs Survey (2013–2015). These data are available through Statistics Canada’s Public Use Microdata Files Collection (https://www150.statcan.gc.ca/n1/pub/11-625-x/11-625-x2010000-eng.htm) and the Data Liberation Initiative (http://dli-idd-nesstar.statcan.gc.ca/webview/).

## Abbreviations

MLA: Minimum legal age
CTUMS: Canadian tobacco use monitoring survey
CTADS: Canadian tobacco alcohol and drugs survey
AIPW: Augmented inverse propensity weighting
MMWS: Marginal mean weighting through stratification
IPW: Inverse probability weighting
