# Supplementary material for: Too young for Cannabis? Choice of minimum legal age for legalized non-medical Cannabis in Canada
Source: BMC Public Health. 2020 May 14;20:557. doi: 10.1186/s12889-020-08639-z (PMC7222435; doi:10.1186/s12889-020-08639-z)
Supplement: Supplementary file 1 — Additional file 1. Supplementary materials. [file 12889_2020_8639_MOESM1_ESM.docx]

**S.1 Study Sample**

Figure A1: Study sample included in base case analysis

Full Sample (CTUMS + CTADS)

N=214,198

Age at survey between 21 and 65 (inclusive)

N=124,924

Ever cannabis users

N=57,524

Age of first cannabis use available

N=55,506

Age of first cannabis use <=24 years N=51,452

Respondents who used cannabis more than once in life N=44,035

Excluded N=89,274

Excluded N=67,400

Excluded N=2,018

Excluded N=4,054

Excluded N=7,417

**S.2 Expanded set of coefficients & full set of contrasts**

Table A1: Full set of coefficients for base case analysis using AIPW

|  | Education (CTUMS) | Current cigarette smoking (CTUMS+CTADS) | Self-reported general health (CTADS) | Self-reported mental health (CTADS) |
| --- | --- | --- | --- | --- |
| **Average Treatment Effect Estimate** | |  |  |  |
| Age of first use 18 years | 0.275*** | -0.094*** | 0.077** | 0.044 |
|  | (0.026) | (0.009) | (0.037) | (0.034) |
| Age of first use 19-20 years | 0.394*** | -0.121*** | 0.057** | 0.086*** |
|  | (0.020) | (0.010) | (0.025) | (0.023) |
| Age of first use 21-24 years | 0.590*** | -0.126*** | 0.110*** | 0.068** |
|  | (0.040) | (0.011) | (0.030) | (0.030) |
| **Potential Outcome Mean Estimate** |  |  |  |  |
| Age of first use <18 years | 3.607*** | 0.394*** | 2.734*** | 2.967*** |
|  | (0.044) | (0.012) | (0.029) | (0.031) |
| **Potential Outcome Equation (Age of first use <18 years)** | | |  |  |
| Age | 0.008*** | -0.003*** | -0.008*** | -0.004*** |
|  | (0.003) | (0.000) | (0.002) | (0.001) |
| Male | -0.356*** | -0.011* | -0.052* | 0.097*** |
|  | (0.046) | (0.006) | (0.031) | (0.035) |
| Urban | 0.440*** | -0.042*** | 0.015 | 0.018 |
|  | (0.035) | (0.010) | (0.033) | (0.034) |
| Widowed/Divorced/Separated | -0.098** | 0.135*** | -0.061** | -0.121*** |
|  | (0.039) | (0.013) | (0.028) | (0.037) |
| Single/Never married | 0.120*** | 0.076*** | -0.081*** | -0.121*** |
|  | (0.025) | (0.008) | (0.031) | (0.037) |
| French | -0.070 | -- | -- | -- |
|  | (0.078) |  |  |  |
| Both English and French | -0.241** | -- | -- | -- |
|  | (0.121) |  |  |  |
| Other languages | -0.226 | -- | -- | -- |
|  | (0.159) |  |  |  |
| Household Size | -0.020** | -0.007* | 0.040*** | 0.028* |
|  | (0.009) | (0.004) | (0.009) | (0.016) |
| Cigarette smoking | -0.793*** | -- | -0.405*** | -0.229*** |
|  | (0.029) |  | (0.016) | (0.040) |
| Other tobacco product use | 0.047* | 0.236*** | -0.013 | 0.008 |
|  | (0.025) | (0.011) | (0.040) | (0.034) |
| Constant | 3.561*** | 0.572*** | 3.074*** | 3.061*** |
|  | (0.124) | (0.014) | (0.076) | (0.064) |
| **Potential Outcome Equation (Age of first use 18 years)** | | |  |  |
| Age | 0.005** | -0.001** | -0.010** | -0.004 |
|  | (0.002) | (0.001) | (0.004) | (0.003) |
| Male | -0.417*** | -0.000 | -0.167*** | 0.036 |
|  | (0.041) | (0.009) | (0.031) | (0.066) |
| Urban | 0.545*** | -0.043*** | -0.017 | 0.075* |
|  | (0.043) | (0.011) | (0.037) | (0.042) |
| Widowed/Divorced/Separated | -0.220*** | 0.113*** | -0.305*** | -0.261** |
|  | (0.080) | (0.018) | (0.059) | (0.103) |
| Single/Never married | 0.107*** | 0.036** | -0.147 | -0.083 |
|  | (0.039) | (0.016) | (0.095) | (0.080) |
| French | -0.234*** | -- | -- | -- |
|  | (0.070) |  |  |  |
| Both English and French | -0.115 | -- | -- | -- |
|  | (0.294) |  |  |  |
| Other languages | -0.229 | -- | -- | -- |
|  | (0.292) |  |  |  |
| Household Size | 0.003 | -0.012** | -0.014 | -0.005 |
|  | (0.013) | (0.005) | (0.036) | (0.034) |
| Cigarette smoking | -0.737*** | -- | -0.357*** | -0.218*** |
|  | (0.060) |  | (0.049) | (0.062) |
| Other tobacco product use | 0.114 | 0.205*** | 0.143 | 0.114 |
|  | (0.084) | (0.018) | (0.097) | (0.124) |
| Constant | 3.732*** | 0.492*** | 3.294*** | 3.167*** |
|  | (0.118) | (0.027) | (0.273) | (0.218) |
| **Potential Outcome Equation (Age of first use 19-20 years)** | | |  |  |
| Age | -0.002 | -0.001*** | -0.011*** | -0.003** |
|  | (0.003) | (0.000) | (0.001) | (0.001) |
| Male | -0.353*** | 0.007 | -0.125*** | -0.035 |
|  | (0.059) | (0.007) | (0.044) | (0.048) |
| Urban | 0.564*** | -0.057*** | -0.015 | -0.078 |
|  | (0.069) | (0.016) | (0.034) | (0.063) |
| Widowed/Divorced/Separated | -0.160** | 0.146*** | -0.027 | -0.067 |
|  | (0.063) | (0.029) | (0.087) | (0.071) |
| Single/Never married | 0.019 | 0.045*** | -0.155*** | -0.103 |
|  | (0.071) | (0.012) | (0.059) | (0.063) |
| French | -0.360** | -- | -- | -- |
|  | (0.144) |  |  |  |
| Both English and French | -0.043 | -- | -- | -- |
|  | (0.260) |  |  |  |
| Other languages | -0.352* | -- | -- | -- |
|  | (0.186) |  |  |  |
| Household Size | -0.015 | -0.013*** | 0.026*** | 0.010 |
|  | (0.018) | (0.004) | (0.008) | (0.022) |
| Cigarette smoking | -0.917*** | -- | -0.361*** | -0.181** |
|  | (0.062) |  | (0.091) | (0.082) |
| Other tobacco product use | 0.117 | 0.202*** | 0.056 | 0.098 |
|  | (0.107) | (0.016) | (0.095) | (0.067) |
| Constant | 4.055*** | 0.411*** | 3.519*** | 3.334*** |
|  | (0.171) | (0.035) | (0.075) | (0.125) |
| **Potential Outcome Equation (Age of first use 21-24 years)** | | |  |  |
| Age | -0.011*** | -0.001 | -0.008*** | -0.002 |
|  | (0.003) | (0.001) | (0.003) | (0.003) |
| Male | -0.300*** | 0.010 | 0.043 | 0.074 |
|  | (0.060) | (0.014) | (0.053) | (0.061) |
| Urban | 0.607*** | -0.004 | 0.071 | 0.023 |
|  | (0.139) | (0.015) | (0.051) | (0.060) |
| Widowed/Divorced/Separated | -0.340*** | 0.145*** | -0.197*** | -0.455*** |
|  | (0.106) | (0.017) | (0.073) | (0.123) |
| Single/Never married | -0.017 | 0.026** | -0.288*** | -0.208** |
|  | (0.070) | (0.013) | (0.039) | (0.086) |
| French | -0.516*** | -- | -- | -- |
|  | (0.173) |  |  |  |
| Both English and French | -0.607 | -- | -- | -- |
|  | (0.520) |  |  |  |
| Other languages | -0.496** | -- | -- | -- |
|  | (0.194) |  |  |  |
| Household Size | -0.024 | -0.002 | -0.005 | 0.014 |
|  | (0.036) | (0.005) | (0.024) | (0.023) |
| Cigarette smoking | -0.693*** | -- | -0.424*** | -0.295** |
|  | (0.066) |  | (0.091) | (0.116) |
| Other tobacco product use | -0.196 | 0.255*** | 0.086 | 0.398*** |
|  | (0.131) | (0.032) | (0.172) | (0.121) |
| Constant | 4.499*** | 0.350*** | 3.309*** | 3.311*** |
|  | (0.251) | (0.042) | (0.168) | (0.199) |
| **Probability of Treatment Equation (Age of first use 18 years)** | | |  |  |
| Age | 0.027*** | 0.026*** | 0.014*** | 0.014*** |
|  | (0.002) | (0.002) | (0.003) | (0.003) |
| Male | 0.178*** | 0.178*** | 0.126* | 0.122* |
|  | (0.022) | (0.026) | (0.073) | (0.074) |
| Urban | 0.064 | 0.093** | 0.152* | 0.158* |
|  | (0.041) | (0.036) | (0.085) | (0.084) |
| Widowed/Divorced/Separated | -0.042 | -0.083* | 0.078 | 0.079 |
|  | (0.060) | (0.049) | (0.133) | (0.132) |
| Single/Never married | 0.153*** | 0.114*** | 0.156 | 0.155 |
|  | (0.029) | (0.032) | (0.121) | (0.121) |
| French | 0.012 | -- | -- | -- |
|  | (0.016) |  |  |  |
| Both English and French | 0.034 | -- | -- | -- |
|  | (0.116) |  |  |  |
| Other languages | -0.013 | -- | -- | -- |
|  | (0.268) |  |  |  |
| Household Size | 0.285*** | 0.018 | 0.005 | 0.005 |
|  | (0.086) | (0.016) | (0.025) | (0.025) |
| Cigarette smoking | -0.441*** | -- | -0.443*** | -0.444*** |
|  | (0.044) |  | (0.070) | (0.068) |
| Other tobacco product use | -0.112** | -0.220*** | -0.175 | -0.172 |
|  | (0.047) | (0.043) | (0.147) | (0.149) |
| Constant | -2.194*** | -2.329*** | -1.609*** | -1.606*** |
|  | (0.100) | (0.102) | (0.205) | (0.205) |
| **Probability of Treatment Equation (Age of first use 19-20 years)** | | |  |  |
| Age | 0.042*** | 0.038*** | 0.018*** | 0.018*** |
|  | (0.003) | (0.002) | (0.002) | (0.002) |
| Male | 0.109** | 0.120*** | 0.093 | 0.092 |
|  | (0.047) | (0.046) | (0.072) | (0.072) |
| Urban | 0.016 | 0.061 | 0.165*** | 0.169*** |
|  | (0.034) | (0.037) | (0.051) | (0.050) |
| Widowed/Divorced/Separated | -0.127*** | -0.178*** | 0.021 | 0.027 |
|  | (0.040) | (0.042) | (0.089) | (0.089) |
| Single/Never married | 0.256*** | 0.164*** | 0.098 | 0.098 |
|  | (0.036) | (0.047) | (0.131) | (0.132) |
| French | -0.038*** | -- | -- | -- |
|  | (0.013) |  |  |  |
| Both English and French | 0.050 | -- | -- | -- |
|  | (0.183) |  |  |  |
| Other languages | 0.088 | -- | -- | -- |
|  | (0.212) |  |  |  |
| Household Size | 0.809*** | -0.019 | 0.017 | 0.018 |
|  | (0.182) | (0.012) | (0.031) | (0.031) |
| Cigarette smoking | -0.555*** | -- | -0.688*** | -0.686*** |
|  | (0.044) |  | (0.118) | (0.119) |
| Other tobacco product use | -0.198*** | -0.315*** | -0.092 | -0.087 |
|  | (0.034) | (0.033) | (0.128) | (0.128) |
| Constant | -2.418*** | -2.580*** | -1.839*** | -1.843*** |
|  | (0.147) | (0.129) | (0.199) | (0.199) |
| **Probability of Treatment Equation (Age of first use 21-24 years)** | | |  |  |
| Age | 0.061*** | 0.052*** | 0.023*** | 0.023*** |
|  | (0.002) | (0.002) | (0.002) | (0.002) |
| Male | -0.134*** | -0.093* | -0.046 | -0.045 |
|  | (0.049) | (0.049) | (0.099) | (0.100) |
| Urban | 0.077* | 0.085*** | 0.055 | 0.065 |
|  | (0.040) | (0.033) | (0.091) | (0.093) |
| Widowed/Divorced/Separated | -0.053 | -0.156*** | -0.214 | -0.210 |
|  | (0.081) | (0.058) | (0.140) | (0.134) |
| Single/Never married | 0.172** | 0.098 | 0.197** | 0.196** |
|  | (0.076) | (0.063) | (0.077) | (0.080) |
| French | -0.140*** | -- | -- | -- |
|  | (0.022) |  |  |  |
| Both English and French | -0.216 | -- | -- | -- |
|  | (0.183) |  |  |  |
| Other languages | 0.142 | -- | -- | -- |
|  | (0.193) |  |  |  |
| Household Size | 0.944*** | -0.116*** | -0.081* | -0.079* |
|  | (0.204) | (0.011) | (0.046) | (0.045) |
| Cigarette smoking | -0.533*** | -- | -0.632*** | -0.627*** |
|  | (0.057) |  | (0.112) | (0.110) |
| Other tobacco product use | -0.273*** | -0.453*** | -0.522*** | -0.521*** |
|  | (0.054) | (0.064) | (0.153) | (0.153) |
| Constant | -3.469*** | -3.452*** | -2.282*** | -2.292*** |
|  | (0.210) | (0.145) | (0.247) | (0.252) |
|  | 35,904 | 42,610 | 6,598 | 6,593 |

Table A2: Full set of coefficients for base case analysis with MMWS

|  | Education (CTUMS) | Current cigarette smoking (CTUMS+CTADS) | Self-reported general health (CTADS) | Self-reported mental health (CTADS) |
| --- | --- | --- | --- | --- |
| **First stage regression** |  |  |  |  |
| *Outcome: Age of first cannabis use* | |  |  |  |
| Age | 0.041*** | 0.036*** | 0.017*** | 0.017*** |
|  | (0.002) | (0.001) | (0.002) | (0.002) |
| Male | 0.057* | 0.069* | 0.046 | 0.046 |
|  | (0.032) | (0.036) | (0.067) | (0.067) |
| Urban | 0.037 | 0.068*** | 0.110*** | 0.110*** |
|  | (0.024) | (0.023) | (0.035) | (0.035) |
| Widowed/Divorced/Separated | -0.052 | -0.115*** | -0.045 | -0.045 |
|  | (0.040) | (0.040) | (0.079) | (0.079) |
| Single/Never married | 0.216*** | 0.147*** | 0.145* | 0.145* |
|  | (0.033) | (0.035) | (0.087) | (0.087) |
| Household size | -0.044*** | -0.030*** | -0.017 | -0.017 |
|  | (0.011) | (0.009) | (0.026) | (0.026) |
| French | -0.033 | -- | -- | -- |
|  | (0.160) |  |  |  |
| Both English and French | 0.077 | -- | -- | -- |
|  | (0.171) |  |  |  |
| Other languages | 0.651*** | -- | -- | -- |
|  | (0.141) |  |  |  |
| Current cigarette smoking | -0.487*** | -- | -0.569*** | -0.569*** |
|  | (0.044) |  | (0.082) | (0.082) |
| Other tobacco product use | -0.165*** | -0.288*** | -0.214** | -0.214** |
|  | (0.035) | (0.030) | (0.088) | (0.088) |
| Pseudo R squared | 0.040 | 0.030 | 0.019 | 0.019 |
| **Second stage regression** |  |  |  |  |
| *Outcome:* | *Education* | *Current cigarette smoking* | *Self-reported general health* | *Self-reported mental health* |
| Age of first use 18 years | 0.283*** | -0.094*** | 0.070 | 0.034 |
|  | (0.030) | (0.008) | (0.041) | (0.037) |
| Age of first use 19-20 years | 0.414*** | -0.123*** | 0.067** | 0.089** |
|  | (0.023) | (0.010) | (0.023) | (0.029) |
| Age of first use 21-24 years | 0.630*** | -0.141*** | 0.093** | 0.029 |
|  | (0.043) | (0.009) | (0.037) | (0.038) |
| R squared | 0.014 | 0.013 | 0.004 | 0.002 |
| N | 35,971 | 42,610 | 6,598 | 6,593 |

Table A3: Full set of contrasts using AIPW

|  | (1) | (2) | (3) | (4) |
| --- | --- | --- | --- | --- |
| Outcome: | Education (CTUMS) | Current cigarette smoking (CTUMS+CTADS) | Self-reported general health (CTADS) | Self-reported mental health (CTADS) |
| AFU 18 years vs. AFU <18 years | 0.275*** | -0.094*** | 0.077** | 0.044 |
|  | (0.026) | (0.009) | (0.037) | (0.034) |
|  | z=10.5, *p*=0.00 | z=-10.3, *p*=0.00 | z=2.1, *p*=0.04 | z=1.3, *p*=0.20 |
| AFU 19-20 years vs. AFU <18 years | 0.394*** | -0.121*** | 0.057** | 0.086*** |
|  | (0.020) | (0.010) | (0.025) | (0.023) |
|  | z=19.9, *p*=0.00 | z=-11.8, *p*=0.00 | z=2.3, *p*=0.02 | z=3.7, *p*=0.00 |
| AFU 21-24 years vs. AFU < 18 years | 0.590*** | -0.126*** | 0.110*** | 0.068** |
|  | (0.040) | (0.011) | (0.030) | (0.030) |
|  | z=14.8, *p*=0.00 | z=-12.0, *p*=0.00 | z=3.6, *p*=0.00 | z=2.2, *p*=0.03 |
| AFU 19-20 years vs. AFU 18 years | 0.119*** | -0.026*** | -0.021 | 0.043 |
|  | (0.024) | (0.007) | (0.045) | (0.043) |
|  | z=4.87, *p*=0.00 | z=-3.86, *p*=0.00 | z=-0.45, *p*=0.65 | z=1.00, *p*=0.32 |
| AFU 21-24 years vs. AFU 18 years | 0.315*** | -0.032*** | 0.033 | 0.024 |
|  | (0.042) | (0.009) | (0.039) | (0.037) |
|  | z=7.42, *p*=0.00 | z=-3.60, *p*=0.00 | z=0.85, *p*=0.40 | z=0.66, *p*=0.51 |
| AFU 21-24 years vs. AFU 19-20 years | 0.196*** | -0.006 | 0.054 | 0.018 |
|  | (0.039) | (0.012) | (0.033) | (0.041) |
|  | z=4.99, *p*=0.00 | z=0.47, *p*=0.64 | z=1.61, *p*=0.11 | z=0.45, *p*=0.65 |

Note: AFU: Age of first use. Data are from CTUMS 2004-2012 and CTADS 2013 and 2015 as indicated in table. Estimates are the difference in outcome for the specific age group of first cannabis use relative to the reference category of age of first use <18 years. Linear regression models were estimated for all outcomes (including ordered outcomes i.e., education, general health and mental health.) All models include province and year fixed effects, as well as controls for: respondent’s age, household size, sex, place of residence (urban/rural), marital status and use of tobacco products other than cigarettes). Models in column 1 also control for language spoken at home. All models except column 2 control for respondent’s smoking status. Sample includes respondents with current age 21-65 years who initiated cannabis before age 25. Only respondents who used cannabis more than once in lifetime are included. Standard errors (in parentheses) are clustered at province level. Significance levels are: *** p<0.01, ** p<0.05, * p<0.1.

Table A4: Full set of contrasts using MMWS

|  | (1) | (2) | (3) | (4) |
| --- | --- | --- | --- | --- |
| Outcome: | Education (CTUMS) | Current cigarette smoking (CTUMS+CTADS) | Self-reported general health (CTADS) | Self-reported mental health (CTADS) |
| AFU 18 years vs. AFU <18 years | 0.283*** | -0.094*** | 0.070 | 0.034 |
|  | (0.030) | (0.008) | (0.041) | (0.037) |
|  | *t*=9.36, *p*=0.00 | *t*=11.47, *p*=0.00 | *t*=1.72, *p*=0.12 | *t*=0.92, *p*=0.38 |
| AFU 19-20 years vs. AFU <18 years | 0.414*** | -0.123*** | 0.067** | 0.089** |
|  | (0.023) | (0.010) | (0.023) | (0.029) |
|  | *t*=17.92, *p*=0.00 | *t*=12.46, *p*=0.00 | *t*=2.90, *p*=0.02 | *t*=3.11, *p*=0.01 |
| AFU 21-24 years vs. AFU < 18 years | 0.630*** | -0.141*** | 0.093** | 0.029 |
|  | (0.043) | (0.009) | (0.037) | (0.038) |
|  | *t*=14.55, *p*=0.00 | *t*=15.33, *p*=0.00 | *t*=2.53, *p*=0.03 | *t*=0.77, *p*=0.46 |
| AFU 19-20 years vs. AFU 18 years | 0.131*** | -0.029*** | -0.002 | 0.055 |
|  | (0.022) | (0.007) | (0.053) | (0.046) |
|  | t=6.04, p=0.00 | *t*=-4.12, *p*=0.00 | *t*=-0.04, *p*=0.97 | *t*=1.21, *p*=0.26 |
| AFU 21-24 years vs. AFU 18 years | 0.346*** | -0.048*** | 0.023 | -0.005 |
|  | (0.044) | (0.009) | (0.052) | (0.046) |
|  | t=7.93, p=0.00 | *t*=-5.08, *p*=0.00 | *t*=0.46, *p*=0.66 | *t*=-0.10, *p*=0.92 |
| AFU 21-24 years vs. AFU 19-20 years | 0.215*** | -0.019 | 0.026 | -0.060 |
|  | (0.043) | (0.011) | (0.049) | (0.052) |
|  | t=5.06, p=0.00 | *t*=-1.62, *p*=0.14 | *t*=0.53, *p*=0.61 | *t*=-1.16, *p*=0.28 |

Note: AFU: Age of first use. Data are from CTUMS 2004-2012 and CTADS 2013 and 2015 as indicated in table. Estimates are the difference in outcome for the specific age group of first cannabis use relative to the reference category of age of first use <18 years. Linear regression models were estimated for all outcomes (including ordered outcomes i.e., education, general health and mental health.) All models include province and year fixed effects, as well as controls for: respondent’s age, household size, sex, place of residence (urban/rural), marital status and use of tobacco products other than cigarettes). Models in column 1 also control for language spoken at home. All models except column 2 control for respondent’s smoking status. Sample includes respondents with current age 21-65 years who initiated cannabis before age 25. Only respondents who used cannabis more than once in lifetime are included. Standard errors (in parentheses) are clustered at province level. Significance levels are: *** p<0.01, ** p<0.05, * p<0.1.

**S.3 Standardized differences with AIPW**

**(a) For education regression**

|  | Raw | Weighted |
| --- | --- | --- |
| Number of observations | 35,904 | 35,904 |
| Age of first use <18 years | 23,064 | 8,956.6 |
| Age of first use 18-19 years | 5,235 | 8,955.5 |
| Age of first use 19-21 years | 5,239 | 8,975.8 |
| Age of first use 21-24 years | 2,366 | 9,016.1 |

|  | **Standardized differences** | |
| --- | --- | --- |
| **Covariate** | **Raw** | **Weighted** |
| *Age of first use 18-19 years* |  |  |
| Age | 0.32 | 0.02 |
| Male | 0.11 | -0.00 |
| Urban | -0.01 | -0.00 |
| Widowed/Divorced/Separated | 0.05 | 0.01 |
| Single/Never married | -0.12 | -0.01 |
| Household size | -0.04 | -0.00 |
| French | -0.08 | -0.00 |
| Both English and French | -0.01 | -0.00 |
| Other languages | 0.00 | -0.00 |
| Cigarette smoking | -0.24 | -0.01 |
| Other tobacco product use | -0.10 | -0.00 |
| *Age of first use 19-21 years* |  |  |
| Age | 0.48 | 0.01 |
| Male | 0.09 | 0.01 |
| Urban | -0.05 | 0.00 |
| Widowed/Divorced/Separated | 0.08 | -0.00 |
| Single/Never married | -0.16 | 0.00 |
| Household size | -0.14 | 0.00 |
| French | -0.09 | -0.02 |
| Both English and French | 0.01 | 0.00 |
| Other languages | 0.04 | 0.00 |
| Cigarette smoking | -0.30 | -0.01 |
| Other tobacco product use | -0.16 | -0.00 |
| *Age of first use 21-24 years* |  |  |
| Age | 0.77 | 0.01 |
| Male | -0.02 | 0.02 |
| Urban | -0.01 | -0.01 |
| Widowed/Divorced/Separated | 0.22 | -0.02 |
| Single/Never married | -0.31 | -0.01 |
| Household size | -0.32 | 0.01 |
| French | -0.15 | 0.00 |
| Both English and French | 0.02 | -0.01 |
| Other languages | 0.03 | -0.01 |
| Cigarette smoking | -0.31 | -0.05 |
| Other tobacco product use | -0.24 | 0.02 |

**(b) For cigarette smoking regression**

|  | Raw | Weighted |
| --- | --- | --- |
| Number of observations | 42,610 | 42,610 |
| Age of first use <18 years | 26,868 | 10,637.3 |
| Age of first use 18-19 years | 6,288 | 10,652.4 |
| Age of first use 19-21 years | 6,462 | 10,662.5 |
| Age of first use 21-24 years | 2,992 | 10,657.8 |

|  | **Standardized differences** | |
| --- | --- | --- |
| **Covariate** | **Raw** | **Weighted** |
| *Age of first use 18-19 years* |  |  |
| Age | 0.30 | 0.01 |
| Male | 0.10 | 0.00 |
| Urban | -0.00 | -0.00 |
| Widowed/Divorced/Separated | 0.05 | 0.00 |
| Single/Never married | -0.11 | -0.00 |
| Household size | -0.05 | 0.00 |
| Other tobacco product use | -0.10 | 0.00 |
| *Age of first use 19-21 years* |  |  |
| Age | 0.45 | -0.00 |
| Male | .08 | 0.01 |
| Urban | -.04 | 0.01 |
| Widowed/Divorced/Separated | .07 | -0.01 |
| Single/Never married | -0.16 | 0.01 |
| Household size | -0.13 | 0.01 |
| Other tobacco product use | -0.16 | -0.00 |
| *Age of first use 21-24 years* |  |  |
| Age | 0.70 | 0.01 |
| Male | -0.02 | 0.01 |
| Urban | -0.02 | -0.00 |
| Widowed/Divorced/Separated | 0.18 | -0.01 |
| Single/Never married | -0.27 | -0.01 |
| Household size | -0.30 | -0.00 |
| Other tobacco product use | -0.24 | 0.00 |

**(c) For general health regression**

|  | Raw | Weighted |
| --- | --- | --- |
| Number of observations | 6,598 | 6,598 |
| Age of first use <18 years | 3,737 | 1,649.7 |
| Age of first use 18-19 years | 1,036 | 1,648.2 |
| Age of first use 19-21 years | 1,208 | 1,648.9 |
| Age of first use 21-24 years | 617 | 1,651.2 |

|  | **Standardized differences** | |
| --- | --- | --- |
| **Covariate** | **Raw** | **Weighted** |
| *Age of first use 18-19 years* |  |  |
| Age | 0.19 | 0.00 |
| Male | 0.05 | 0.00 |
| Urban | 0.04 | -0.01 |
| Widowed/Divorced/Separated | 0.04 | -0.00 |
| Single/Never married | -0.03 | 0.01 |
| Household size | -0.07 | 0.00 |
| Cigarette smoking | -0.21 | 0.00 |
| Other tobacco product use | -0.10 | -0.00 |
| *Age of first use 19-21 years* |  |  |
| Age | 0.27 | 0.01 |
| Male | 0.04 | -0.00 |
| Urban | 0.04 | 0.00 |
| Widowed/Divorced/Separated | 0.04 | 0.00 |
| Single/Never married | -0.09 | -0.00 |
| Household size | -0.07 | -0.00 |
| Cigarette smoking | -0.32 | -0.00 |
| Other tobacco product use | -0.11 | -0.01 |
| *Age of first use 21-24 years* |  |  |
| Age | 0.36 | 0.02 |
| Male | -0.03 | -0.01 |
| Urban | -0.01 | 0.01 |
| Widowed/Divorced/Separated | 0.01 | 0.02 |
| Single/Never married | -0.05 | -0.04 |
| Household size | -0.20 | 0.01 |
| Cigarette smoking | -0.31 | 0.02 |
| Other tobacco product use | -0.22 | 0.01 |

**(d) For mental health regression**

|  | Raw | Weighted |
| --- | --- | --- |
| Number of observations | 6,593 | 6,593 |
| Age of first use <18 years | 3,735 | 1,648.6 |
| Age of first use 18-19 years | 1,036 | 1,647.3 |
| Age of first use 19-21 years | 1,205 | 1,647.9 |
| Age of first use 21-24 years | 617 | 1,649.2 |

|  | **Standardized differences** | |
| --- | --- | --- |
| **Covariate** | **Raw** | **Weighted** |
| *Age of first use 18-19 years* |  |  |
| Age | 0.19 | 0.00 |
| Male | 0.05 | 0.00 |
| Urban | 0.04 | -0.01 |
| Widowed/Divorced/Separated | 0.04 | -0.00 |
| Single/Never married | -0.03 | 0.01 |
| Household size | -0.06 | 0.00 |
| Cigarette smoking | -0.21 | 0.00 |
| Other tobacco product use | -0.10 | -0.00 |
| *Age of first use 19-21 years* |  |  |
| Age | 0.27 | 0.01 |
| Male | 0.04 | -0.00 |
| Urban | 0.04 | 0.00 |
| Widowed/Divorced/Separated | 0.04 | 0.00 |
| Single/Never married | -0.09 | -0.00 |
| Household size | -0.07 | -0.00 |
| Cigarette smoking | -0.32 | -0.00 |
| Other tobacco product use | -0.11 | -0.01 |
| *Age of first use 21-24 years* |  |  |
| Age | 0.36 | 0.02 |
| Male | -0.03 | -0.01 |
| Urban | -0.00 | 0.01 |
| Widowed/Divorced/Separated | 0.01 | 0.02 |
| Single/Never married | -0.05 | -0.04 |
| Household size | -0.20 | 0.01 |
| Cigarette smoking | -0.31 | 0.01 |
| Other tobacco product use | -0.22 | 0.01 |

**S.4 Regression results for alternative study samples**

Table A3. Regression results based on other study samples:

|  | (1) | (2) | (3) | (4) |
| --- | --- | --- | --- | --- |
| Outcome: | Education (CTUMS) | Current cigarette smoking (CTUMS+CTADS) | Self-reported general health (CTADS) | Self-reported mental health (CTADS) |
| ***Panel A: Study sample includes both one-time and more than one time cannabis users*** | | | |  |
| Age of first use 18 years | 0.237*** | -0.098*** | 0.077** | 0.045 |
|  | (0.019) | (0.007) | (0.037) | (0.034) |
| Age of first use 19-20 years | 0.386*** | -0.130*** | 0.055** | 0.087*** |
|  | (0.020) | (0.009) | (0.025) | (0.024) |
| Age of first use 21-24 years | 0.576*** | -0.136*** | 0.110*** | 0.068** |
|  | (0.046) | (0.007) | (0.030) | (0.030) |
| N | 43,059 | 49,792 | 6,606 | 6,601 |
| ***Panel B: Study sample includes only respondents who reported both ‘used cannabis more than once’ and ‘used cannabis in past 12 months’*** | | | |  |
| Age of first use 18 years | 0.283*** | -0.132*** | 0.125** | 0.007 |
|  | (0.079) | (0.017) | (0.055) | (0.061) |
| Age of first use 19-20 years | 0.303*** | -0.163*** | 0.079** | 0.066 |
|  | (0.045) | (0.021) | (0.035) | (0.048) |
| Age of first use 21-24 years | 0.637*** | -0.195*** | 0.081 | 0.054 |
|  | (0.114) | (0.014) | (0.095) | (0.080) |
| N | 11,290 | 13,530 | 2,128 | 2,125 |

Note: Data are from CTUMS 2004-2012 and CTADS 2013 and 2015 as indicated in table. All estimates are based on AIPW method. . Estimates are the difference in outcome for the specific age group of first cannabis use relative to the reference category of age of first use <18 years. Linear regression models were estimated for all outcomes (including ordered outcomes i.e., education, general health and mental health as the Stata command ‘teffects aipw’ for AIPW does not allow use of ordered logit or ordered probit outcome models.) All models include province and year fixed effects, as well as controls for: respondent’s age, household size, sex, place of residence (urban/rural), marital status and use of tobacco products other than cigarettes). Models in column 1 also control for language spoken at home. All models except column 2 control for respondent’s smoking status. Sample includes respondents with current age 21-65 years who initiated marijuana before age 25. Standard errors (in parentheses) are clustered at province level. Significance levels are: *** p<0.01, ** p<0.05, * p<0.1.
